# Rationally Attenuated Vaccines for Venezuelan Equine Encephalitis Protect Against Epidemic Strains with a Single Dose

**DOI:** 10.3390/vaccines8030497

**Published:** 2020-09-02

**Authors:** Shannan L. Rossi, Kasi E. Russell-Lodrigue, Kenneth S. Plante, Nicholas A. Bergren, Rodion Gorchakov, Chad J. Roy, Scott C. Weaver

**Affiliations:** 1 Department of Pathology and Microbiology and Immunology, Institute for Human Infection and Immunity, University of Texas Medical Branch, Galveston, TX 77555, USA; 2 Tulane National Primate Research Center, Covington, LA 70433, USA; kerussel@tulane.edu (K.E.R.-L.); croy@tulane.edu (C.J.R.); 3 Department of Microbiology and Immunology and World Reference Center for Emerging Viruses and Arboviruses, University of Texas Medical Branch, Galveston, TX 77555, USA; ksplante@utmb.edu; 4 Department of Pathology, University of Texas Medical Branch, Galveston, TX 77555, USA; nicholasbergren@gmail.com; 5 Department of Health, Safety and Environment, King Abdullah University of Science and Technology, Thuwal 23955, Saudi Arabia; rodion.gorchakov@kaust.edu.sa; 6 Department of Microbiology and Immunology, Tulane School of Medicine, New Orleans, LA 70112, USA; 7 World Reference Center for Emerging Viruses and Arboviruses, University of Texas Medical Branch, Galveston, TX 77555, USA; 8 Institute for Human Infection and Immunity, University of Texas Medical Branch, Galveston, TX 77555, USA; 9 Department of Microbiology and Immunology, University of Texas Medical Branch, Galveston, TX 77555, USA

**Keywords:** Venezuelan equine encephalitis virus, vaccine, internal ribosome entry site, primates

## Abstract

Venezuelan equine encephalitis virus (VEEV) is a re-emerging virus of human, agriculture, and bioweapon threat importance. No FDA-approved treatment is available to combat Venezuelan equine encephalitis in humans, prompting the need to create a vaccine that is safe, efficacious, and cannot be replicated in the mosquito vector. Here we describe the use of a serotype ID VEEV (ZPC-738) vaccine with an internal ribosome entry site (IRES) to alter gene expression patterns. This ZPC/IRES vaccine was genetically engineered in two ways based on the position of the IRES insertion to create a vaccine that is safe and efficacious. After a single dose, both versions of the ZPC/IRES vaccine elicited neutralizing antibody responses in mice and non-human primates after a single dose, with more robust responses produced by version 2. Further, all mice and primates were protected from viremia following VEEV challenge. These vaccines were also safer in neonatal mice than the current investigational new drug vaccine, TC-83. These results show that IRES-based attenuation of alphavirus genomes consistently produce promising vaccine candidates, with VEEV/IRES version 2 showing promise for further development.

## 1. Introduction

Venezuelan equine encephalitis (VEE) is a re-emerging disease of equids and people with a long history of explosive, mosquito-borne outbreaks as well as endemic disease. Major outbreaks occur when equine-amplification-competent strains emerge from enzootic, sylvatic progenitors that circulate among rodents transmitted by arboreal mosquitoes [1]. These epizootics/epidemics, typically involving subtype IAB and IC strains of VEE virus (VEEV) occur periodically when enzootic, subtype ID strains mutate to gain the equine-amplification-competent and equine-virulent phenotypes [2]. These subtypes are primarily based upon similar serological differences but also upon their ability to cause epidemics. The last major outbreak occurred in Venezuela and Colombia in 1995, affecting approximately 100,000 people and large numbers of equids [3]. However, an estimated 10,000 human cases also occur annually via endemic spillover from the rodent-mosquito cycles that occur in forests, but also periurban areas throughout much of Latin America [4,5].

Unlike many arboviral diseases, VEE typically causes symptomatic infection, ranging from a nonspecific flu-like illness that resolves in about one week to a more severe form seen in 5–15% of cases that involves the central nervous system, especially in children. This severe form is often accompanied by permanent neurologic sequelae or death, and infection also leads to severe immunosuppression. Overall case-fatality rates are typically less than 1%, but teratogenic effects also result in many stillborn deaths during outbreaks [6]. In addition to being a deadly re-emerging virus, VEEV is also a highly efficient biological weapon capable of disabling large populations due to its high degree of stability when delivered as an aerosol and its high degree of infectivity via the respiratory route. It was therefore highly developed by both the United States and the former Soviet Union during the cold war [7].

**Figure 1:**
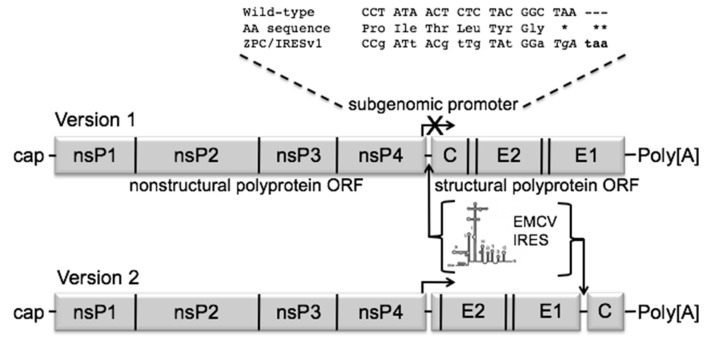
Schematic diagram of the ZPC/IRES vaccine constructs. The subgenomic promoter ablation mutations are shown as lower-case letters. The corresponding amino acid sequence confirming noncoding mutations is shown between the wild type and mutant sequences. The inserted stop codon is shown in bold. * indicates stop codon mutated from ochre to opal. ** indicates an inserted ochre stop codon. Genomic schematics of versions 1 and 2 show the locations of the IRES insertion in lieu of the subgenomic promoter or just upstream of the capsid gene, respectively.

Despite its importance as a re-emerging and biothreat virus, no licensed products exist to prevent or treat VEEV infection. Although experimental vaccines have been developed for many decades, only three have been used in clinical trials. The attenuated TC-83 strain of VEEV, developed by serial cell culture passage of the epidemic Trinidad donkey (TRD) strain [8], has been used as an Investigational New Drug (IND) product in humans for several decades and both live forms and inactivated forms have been used for nearly 50 years in humans and equids (licensed for the latter). Although considered highly effective in equids [9,10], TC-83 is reactogenic in humans and a single dose often fails to induce seroconversion, requiring a boost with an inactivated vaccine form called C-84 [11]. Vaccines report a variety of adverse events, some severe, like rapid fever onset, headache, photophobia. TC-83 also has a number of exclusion criteria, including having egg allergies, being pregnant, or having a first relative diagnosed with diabetes. Interestingly, alternations in glucose metabolism have been modeled in TC-83 vaccinated Golden Syrian hamsters [12] and rhesus macaques [13]. Due to the high level of severe adverse events, TC-83 is only used for laboratory scientists at high risk of infection. The reactogenicity of TC-83 probably results from the reliance on only two point mutations for attenuation [14]. The second vaccine to enter clinical trials, called strain V3526, was a rationally attenuated version of the TRD strain with a PE2 viral polyprotein cleavage-signal mutation, combined with a second-site suppressor mutation [15] (Figure 1). This vaccine, although more stably attenuated than TC-83 [16], also proved reactogenic in a Phase 1 clinical trial, with adverse events including headache, fever, malaise, and sore throat [17].

More recently, a DNA vaccine that expresses codon-optimized versions of the E3-E2-6K-E1 structural protein genes of VEEV strain TRD was developed. This vaccine induces high levels of neutralizing antibodies in rodents and nonhuman primates and protects against VEEV challenge [18]. In a Phase 1 trial, 2–3 doses of the DNA administered intramuscularly or intradermally with an electroporation device were well-tolerated, and induced neutralizing antibodies responses in volunteers receiving low or high doses [19].

Although the DNA vaccine described above could be useful in some situations, the requirement for multiple doses is not optimal for controlling an explosive arboviral outbreak or for responding to a biothreat situation. We therefore have focused mainly on live-attenuated vaccines for VEE, starting with chimeric alphaviruses [20], and later progressing to attenuation via alterations to the expression patterns of the alphavirus open reading frames (Figure 1). These include: (1) inactivation of the subgenomic promoter to eliminate the molar excess of subgenomic RNA that results in high levels of structural protein expression, combined with the introduction of a picornavirus (encephalomyocarditis virus) internal ribosome entry site (IRES) to allow for translation of the structural polyprotein from genomic RNA (Version 1), and; (2) translocation of the capsid protein gene to a separate open reading frame downstream of the envelope glycoprotein genes and behind the IRES to downregulate expression of capsid only (Version 2). This approach was first used with the TC-83 live-attenuated VEE vaccine strain [21]. The subgenomic RNA was eliminated as expected, and mosquito infectivity was also eliminated because the IRES is not efficiently recognized by insect ribosomes. This Version 2 of VEEV/IRES was slightly more immunogenic [22].

Although the original IRES-based attenuation was not successful in terms of immunogenicity using the attenuated TC-83 backbone, the application of this approach to wild-type VEEV strains demonstrated a high degree of attenuation, immunogenicity, and efficacy. Starting with an enzootic subtype IE VEEV strain that represents the cause of extensive endemic human VEE in Mexico and Central America [4,23], IRES-based, live-attenuated vaccine candidates proved highly attenuated and immunogenic after a single immunization of mice, and provided full protection against lethal challenge [24]. Version 1 of this vaccine produced no detectable disease in cynomolgus macaques (*Macaca fascicularis*), the preferred nonhuman primate model for VEE, and protected against aerosol challenge including fever and viremia (this nonhuman primate model, like most human infections, is not lethal) [25].

Although VEEV subtype IE is an important human and sometimes also an equine pathogen [26], its southern distribution ends in western Panama, where enzootic subtype ID occurs southward through eastern Panama, Venezuela, Colombia, Ecuador, Peru, and Bolivia [4]. Furthermore, subtype ID strains are the progenitors of subtype IAB and IC strains, which are responsible for all major epizootics/epidemics ever documented [27]. There is also significant antigenic divergence between subtypes ID and IE, as reflected in weak cross-neutralizing antibody titers after infection or vaccination [28,29]. Furthermore, although the subtype IAB-based V3526 vaccine strain protects mice against aerosol challenge with a subtype IE VEEV strain, it did not significantly limit challenge virus replication [29].

Based on the potential limitations of a subtype IE-based vaccine to protect against subtypes IAB, IC, and ID, we developed additional VEE vaccines based on these same IRES-based attenuation approaches but using ID strain ZPC738 [30], a close relative of subtypes IAB and IC [31,32]. Here, we describe the safety, immunogenicity and efficacy of these vaccines as determined in mice and cynomolgus macaques.

## 2. Materials and Methods

### 2.1. Cell Lines and Viruses

Vero-76 cells were maintained in Dulbecco’s Minimal Essential Media (DMEM) supplemented with 5% fetal bovine serum (FBS) and 1% penicillin/streptomycin (P/S) within a 37 °C incubator containing 5% CO_2_. All viruses used in this study were rescued from infectious cDNA clones. ID ZPC-738 VEEV [32] and IC 3908 VEEV [33] were rescued from cDNA clones as described below.

### 2.2. Creating the IRES Vaccines and Rescue

Creating the ZPC/IRES vaccines was done by modifying the original cDNA clone of the ZPC full-length infectious clone pMI-738 using standard molecular techniques as previously descried [24]. For ZPC/IRESv1, site-directed mutagenesis of the nsP4-subgenomic promoter region was done using a fusion PCR reaction conducted with the following primer set: 5′gATtACgtTgTAtGGaTgAtaaAACGTTACTGGCCGAAGCCGCTTGGAA3′ and 5′TTttaTcAtCCaTAcAacGTaATcGGGGCCCCTCTCAGGTAGCTGAAT3′. The lower-case letters denote mutations. The EMCV IRES sequence was obtained from the VEEV TC-83/IRESv1 clone previously made [21,22]. Naturally occurring SwaI and AflII sites were used to insert the mutated subgenomic promoter and IRES into the pMI-738 cDNA clone. ZPC/IRESv2 was created by fusing the EMCV IRES between the E1 and capsid genes. A TAA stop codon was inserted immediately after E1 followed by a short 3′ UTR sequence upstream of the start of the IRES sequence. A start codon was inserted immediately before the capsid gene. Naturally occurring AvrII, NdeI, and NotI restriction sites in the pMI-738 cDNA were used to assemble the fusion PCR fragments. ZPC/IRES vaccines with individual mutations were also engineered using similar methods using plasmids encoding the desired mutation.

All ZPC/IRES vaccine cDNA clones were fully sequenced by Sanger sequencing and the contigs were assembled into a consensus sequence to ensure only the inserted sequences were present. Viral RNA was obtained by in vitro transcription using an SP6 promoter (mMessage mMachine SP6 transcription kit, Invitrogen, Carlsbad, CA, USA) on a NotI-linearized cDNA template. Vero cells were electroporated with viral RNA as previously described [24]. Supernatant was collected when cytopathic effects were observed and was clarified by centrifugation prior to aliquoting samples and storing at −80 °C.

### 2.3. Titrations

VEEV virus and vaccine titrations were done as previously described [24]. Briefly, Vero cell monolayers in 6- or 12-well plates were infected with serial dilutions of virus supernatant or serum in DMEM supplemented with 2% FBS and 1% P/S. After 1-hour adsorption, monolayers were overlaid with dilution media supplemented with 0.4% agarose and incubated in a 37 °C incubator containing 5% CO_2_. Monolayers were fixed with formalin and plaques were visualized using a crystal violet stain after removing the agarose overlay. Plaques were counted in each well and titers are reported as plaque forming units (pfu)/milliliter (mL).

### 2.4. Plaque Reduction Neutralization Tests

Plaque reduction neutralization tests were performed using sera from vaccinated mice as described in Rossi et al. [24]. Briefly, samples were diluted 1/10 in PBS prior to heat inactivation at 56 °C for 1 h. Serial two-fold dilutions of sera were performed in media prior to adding 800 pfu of virus. After incubating the sera and viruses for an hour at 37 °C, the mixture was added to a monolayer of Vero cells for hour. An overlay containing 0.4% agarose in media was added to each well and incubated for 48 h. Plaques were visualized by crystal violet staining. Average un-neutralized plaque counts varied between 24 and 35, from which the 50% (PRNT_50_) and 80% (PRNT_80_) neutralization levels were calculated.

### 2.5. Replication Curves

Semiconfluent Vero monolayers in T25 flasks were infected with either TC-83, ZPC-738, ZPC/IRESv1, or ZPC/IRESv2 at a multiplicity of infection (MOI) equal to 0.1 in triplicate. After 1 h, the infection was washed thrice with cell culture media and a sample was taken to determine the level of residual virus after infection. At 6, 12, 24, and 48 h post infection, a small sample was removed from the flask and frozen at −80 °C. To create the growth curve, the initial inoculum and all samples were titrated on Vero monolayers in 12-well plates [24].

### 2.6. Serial Passage in Vero Cells

ZPC/IRESv1 was serially passaged in triplicate in T25 flasks of Vero cells at an MOI of 0.1 Forty-eight hours after infection, each replicate was titrated prior to reseeding a new set of Vero cells into a T25 at an MOI of 0.1 to initiate the next passage. At the end of 10 passages, viral RNA was isolated following TRIzol (Invitrogen, Carlsbad, CA, USA) extraction of supernatant. The entire coding region was Sanger sequenced to identify consensus mutations. Mutations observed in the E2 protein, either alone or together, were incorporated into the ZPC/IRESv1 and ZPC/IRESv2 cDNAs.

### 2.7. Mouse Studies

All animal handling was conducted at UTMB in accordance with the UTMB Institutional Animal Care and Use Committee approval (IACUC #0209068). Female CD1 mice were purchased from Charles River Laboratories (Wilmington, MA, USA). Adult mice (aged 6–8 weeks) were used for vaccination and immunogenicity studies similar to previous studies [24]. For vaccination, mice were anesthetized with inhaled isoflurane, then injected subcutaneously in the scruff of the back with 100 μL of virus diluted in DMEM supplemented with 2% FBS and 1% P/S. Vaccination dose was confirmed by back titration of inocula to be 1 × 10^5^ pfu/mouse. Challenge was achieved by delivering VEEV 3908 strain diluted in PBS either subcutaneously (1 × 10^5^ pfu/mouse) or intranasally (1 × 10^4^ pfu, anesthetized mice aspirated 20 μL of virus through the nares). Following vaccination and challenge, mice were observed daily for signs of illness, which included lethargy, ruffled fur, hunched posture, and anorexia. Individuals’ weights were also measured to quantify illness. Any mouse that exhibited signs of neurological infection, including tremors, failure to right and partial hindlimb paralysis, or were unable to reach food or water were humanely euthanized. Euthanized animals were recorded as having died the following day.

To acquire serum to determine viremia or neutralizing antibody levels, blood was collected from anesthetized mice by retro-orbital puncture with a heparinized micro-hematocrit capillary tube (Fisher Scientific, Pittsburgh, PA, USA). In some cases, blood was collected into microfuge tubes containing 225 μL of PBS to achieve a 1:10 dilution of plasma. Blood was centrifuged for 5 min at 3380× *g*, then diluted plasma was removed and frozen at −80 °C until needed.

Neurovirulence safety studies were done in six-day-old CD1 pups from litters randomized among dams. These pups were inoculated intracranially with 20 μL of virus at a titer of 1 × 10^4^ pfu/mouse, and monitored daily for survival.

### 2.8. Nonhuman Primate Studies

Nonhuman primate (NHP) cynomologous macaques (*Macaca fascicularis*) were housed at Tulane National Primate Research Center (TNPRC), which is an AAALAC-accredited facility. All work was approved by the Tulane Institutional Animal Care and Use Committee (Protocol P0171).

Healthy NHPs weighing between 3–6 kg were screened and free of prior infection against alphaviruses (Venezuelan and eastern equine encephalitis viruses, Sindbis, Semliki Forest, and chikungunya viruses) as well as simian immunodeficiency virus, simian type D retrovirus and simian T-lymphotropic virus. NHPs were housed in open metal caging allowing visual contact with others in the room. A standard primate chow and fresh fruits and vegetables were provided daily. An intramuscular injection of ketamine hydrochloride for anesthetization was given prior to collecting blood samples or vaccination.

Prior to the start of the study, some NHPs were implanted with a telemetric device as previously described (Konigsberg Instruments, Pasadena, CA, USA) [25]. Buprenorphine was given during and after the procedure for analgesia. Post-surgery, NHPs were monitored for signs of infection, lethargy, anorexia, dehydration, and device rejection. Core body temperature was recorded wirelessly and reported in one-hour observation intervals. Baseline temperatures were recorded for 6 days prior to the aerosol challenge. Baseline data over a 24-h period were averaged to determine the temperature threshold and deviations were recorded as 1.5 times higher or lower than the standard deviation for each time. Each NHP served as its own temperature control through the use of collected pre-exposure baseline data. NHPs that were not monitored telemetrically had rectal temperatures taken only daily when anesthetized during a procedure.

NHPs assigned to the control group (N = 2) were given saline and those assigned the vaccine group (N = 5) were given 1 × 10^5^ pfu of ZPC/IRESv1, both in the upper deltoid with a single subcutaneous injection of 100 μL. No signs of disease and distress were noted following vaccination. Blood samples were taken by venipuncture on days 1, 10, 23, and 35 to determine neutralizing antibody levels. Sera were clarified from this blood by centrifugation to determine PRNT titer.

Aerosol exposure to VEEV 3908 was performed on day 35 post vaccination. A head-only 16-liter dynamic inhalation aerosol exposure was used to challenge each NHP as previously reported [34]. Collision and AGI samples were used to determine the infectious dose received by each NHP, which fell in the range between 8 × 10^5^ and 9 × 10^6^ pfu. Animals were observed for signs of illness and telemetrically monitored for temperature changes. Blood was taken for the first 3 days following challenge to determine viremia. On day 24 post challenge, NHPs were euthanized by a pre-dose of ketamine hydrochloride followed by an overdose of sodium pentobarbital. Necropsies were performed to determine any gross pathological changes.

### 2.9. Statistical Analyses

All graphs and statistical analyses were created and performed using GraphPad software v8.0. Significance in growth curve titers was determined by a two-way ANOVA with Tukey posthoc tests to compare multiple groups. Vaccine viremia and NHP fever-hours were compared individually using Student’s *t*-test. For values below or above the LOD, one-half of the limit of detection (LOD) was recorded and statistics were conducted. Survival significance was determined by Log-rank (Mantel-Cox) test comparing two groups.

## 3. Results

### 3.1. Creation and Characterization of VEEV ZPC/IRES Vaccines

Based on the success of other IRES-containing alphavirus vaccines [24,35,36], the ID VEEV strain was chosen for additional characterization. The parental pMI-738 infectious clone encoding the ZPC-738 virus was chosen as it is well characterized in vitro and in vivo [32]. Two variations of the ZPC/IRES vaccine were constructed using standard molecular cloning techniques, varying in the functionality of the subgenomic promoter and location of the inserted IRES sequence (Figure 1). In ZPC/IRESv1 (Version 1), the subgenomic promoter was inactivated with the maximum possible number of synonymous mutations while preserving the amino acid sequence of the essential nsP4 protein. An additional stop mutation was also engineered into the construct. This eliminated production of subgenomic RNA, which is normally synthesized in molar excess compared to the genomic RNA. An IRES from encephalomyocarditis virus was inserted between the open reading frames (ORFs) to allow for translation of the structural proteins from the genomic RNA. In ZPC/IRESv2 (Version 2), the subgenomic promoter was left intact for efficient production of the subgenomic RNA transcript encoding the structural genes. The highly immunogenic envelope glycoproteins E3-E1 were not manipulated. Rather, the IRES sequence was inserted between E1 and capsid genes and capsid was shifted to the end of the genome to prevent its expression in insect cells.

**Figure 2:**
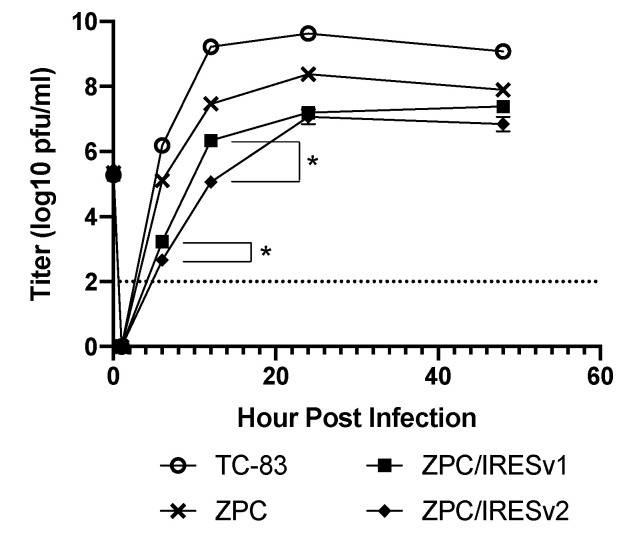
Log_10_-transformed in vitro replication curves. ZPC/IRES vaccine versions 1 and 2 (square and diamond, respectively), ZPC-738 (X) or TC-83 vaccine (open circles) were amplified in Vero cells in triplicate at an MOI of 0.1. Error bars denote standard error. Titers between ZPC/IRESv1 and ZPC/IRESv2 were compared and significant differences are denoted by an asterisk (*, 2-way ANOVA with Tukey multiple comparisons post-hoc test).

To determine the replication kinetics of the ZPC/IRES vaccines and ensure the IRES insertion did not render a replication-defective vaccine, Vero cells were infected at multiplicity of infection (MOI) equal to 0.1 along with the TC-83 and ZPC-738 controls. TC-83 replicated to the highest titers at all timepoints tested significantly higher than all other groups with the exception of immediately after infection (1 h post infection [hpi], Figure 2). ZPC-738 amplified to high titers but about 10-fold lower than TC-83, with a peak of 8.3 log_10_ pfu/mL at 24 hpi. By 48 hpi, there was no statistical difference between ZPC-738, ZPC/IRESv1, or ZPC/IRESv2. At all timepoints tested, IRES vaccines replicated to lower levels than either TC-83 or ZPC-738 with max titers of approximately 7.3 log_10_ pfu/mL. The only statistical differences between ZPC/IRESv1 and ZPC/IRESv2 were noted at 6 hpi (*p* < 0.05) and 12 hpi (*p* < 0.05).

### 3.2. VEEV ZPC/IRES Are Immunogenic and Protective in Mice

**Figure 3:**
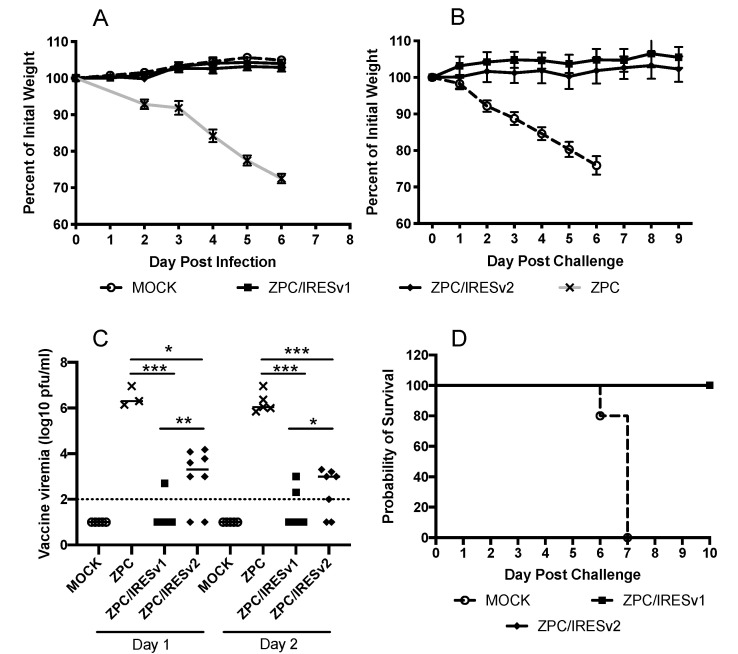
Vaccine efficacy in adult CD1 mice (**A**,**B**) Percent weight change following injection or vaccination or lethal challenge with VEEV 3908 from a representative experiment. The key for panels (**A**,**B**) are the same. (**C**) Viremia following virus infection or vaccination. Serum from blood collected on days 1 or 2 was analyzed and data from 2 independent experiments are combined. Significance directly comparing two groups was determined by unpaired *t*-tests; * *p* > 0.5, ** *p* > 0.01, *** *p* > 0.001. The solid line grouping with the data points represents the mean. The dashed line represents the assay limit of detection. (**D**) Survival in vaccinated groups following lethal challenge with VEEV 3908 from a representative experiment.

To determine the immunogenicity in vivo, adult CD1 mice, which are commonly used for VEEV vaccine testing, were used [24]. Mice were injected subcutaneously with 1 × 10^5^ pfu of virus or vaccine and monitored for loss of weight or signs of neurovirulence. Weights were measured daily as they directly correlate with disease in this model; the only weight loss was measured in VEEV ZPC-infected mice (Figure 3A). Blood was taken on days 1 and 2 post infection to determine viremia levels. While ZPC-738 generated a high viremia on days 1 and 2, the ZPC/IRES vaccines produced significantly lower viremia (Figure 3B, *p* > 0.001). Only 1 of 10 mice showed a low-level viremia on days 1 and 2, in 10 mice on day 2. The viremia detected in ZPC/IRESv2-vaccinated mice was significantly higher on both days 1 (*p* = 0.001) and 2 (*p* = 0.027) compared to ZPC/IRESv1-vaccinated mice, and occurred in a higher percentage in the group (6 of 8 on day 1, and 5 of 7 on day 2). Mice were subcutaneously challenged with 1 × 10^5^ pfu of the lethal subtype IC strain 3908, isolated from a 1995 human case during an epidemic in Venezuela [3], 4 weeks after a single vaccination. All mice retained their weight (Figure 3C), and failed to show any signs of disease and survived (Figure 3D). In contrast, mock-vaccinated mice readily succumb to lethal infection following by a steady loss in weight, resulting in euthanasia based upon humane endpoints.

**Figure 4:**
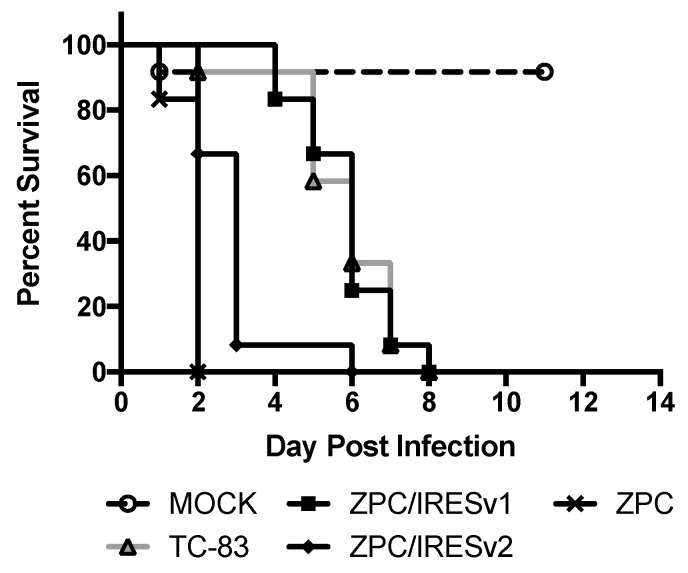
Survival in vaccinated mice following lethal intranasal 3908 challenge.

Alphaviruses like VEEV that cause neurovirulent disease are often also highly infectious by the respiratory route. To test whether the ZPC/IRES vaccine candidates can also protect against this highly lethal route, adult CD1 mice were vaccinated with 1 × 10^5^ pfu subcutaneously and challenged intranasally with 1 × 10^4^ pfu of 3908 one month later (Figure 4). Similar to a subcutaneous challenge, all vaccinated mice were fully protected whereas mock-vaccinated mice succumbed to disease between days 7 and 8 post-challenge.

**Figure 5:**
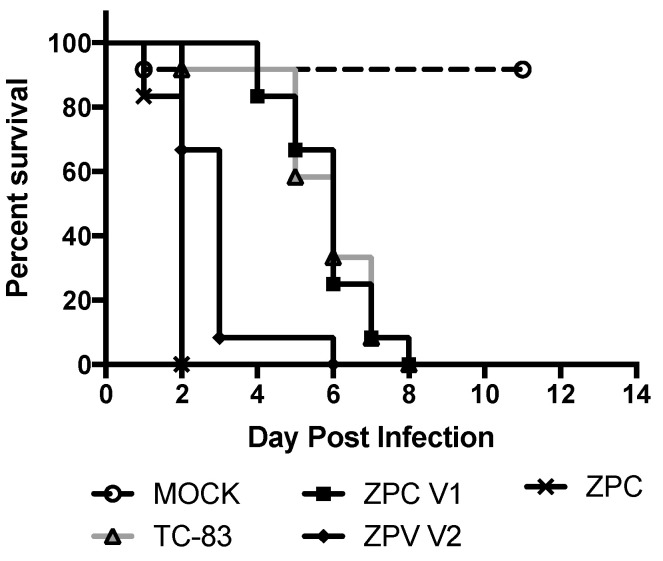
Survival following intracranial injection in neonatal murine pups.

In order to differentiate further the attenuation between ZPC/IRESv1 and ZPC/IRESv2, murine neonatal neurovirulence and safety testing was performed. VEEV viruses are known to be neurovirulent in neonates and any delay or lack of death is a measure of attenuation. Six-day-old pups were injected intracranially with ZPC/IRES vaccines, PBS, TC-83, or ZPC-738, and survival was recorded (Figure 5). As expected, no pups (0/12) survived ZPC-738 infection with uniform lethality occurring on 2 dpi. Surprisingly, ZPC/IRESv2 also caused uniform lethality with all but one pup succumbing by day 3 (0/12). Despite the curve similarity, there was a significant difference in the timing of mortality between ZPC-738 and ZPC/IRESv2 (*p* > 0.001). Another clustering of survival occurred between TC-83 and ZPC/IRESv1. Although no pups in either vaccination group survived, there was no statistical difference between these curves, indicating that ZPC/IRESv1 has a neurovirulence profile similar to the IND vaccine TC-83. One of the twelve pups injected with PBS died on day 1, presumably the result of the inoculation procedure.

### 3.3. In Vitro Stability

To assess the stability of the ZPC/IRES vaccine candidates, ZPC/IRESv1 was passaged serially 10 times in Vero cells in three replicates at low multiplicity (MOI = 0.1), and then sequenced by RT-PCR amplification followed by Sanger sequencing to detect changes in the consensus sequence. Each replicate gained 6 consensus mutations in the nsP2, snP4 (both synonymous), E2, and E1 genes. These mutations were nonsynonymous where two uncharged amino acids were replaced with arginine residues. One partial mutation (resulting in a mixed SNP) in the IRES sequence was also observed (Table 1).

**Figure 6:**
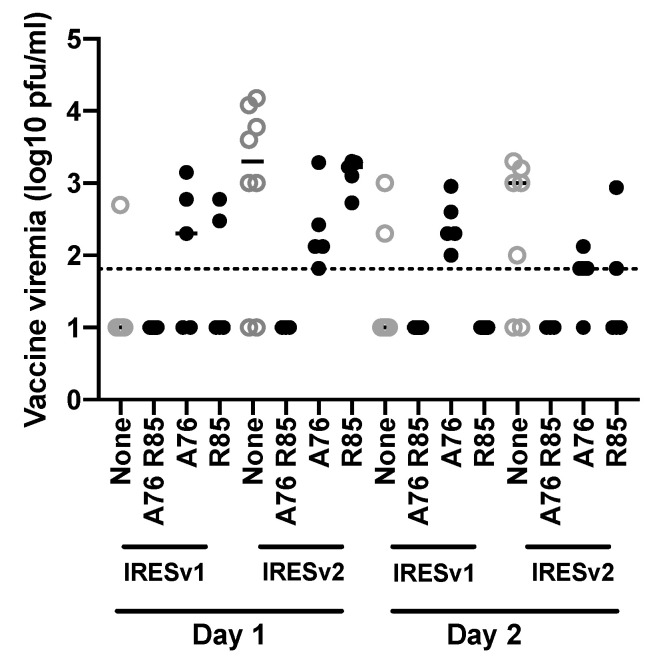
Viremia following vaccination with mutated ZPC/IRES vaccines. Individual data points are shown in log_10_-transformed titers. The mean is denoted by a bar in each group. ZPC/IRESv1 and ZPC/IRESv2 are included as grayed out open circles as a comparison from data in Figure 3B. Dashed line denotes limit of detection = 1.81 log_10_ pfu/mL.

**Figure 7:**
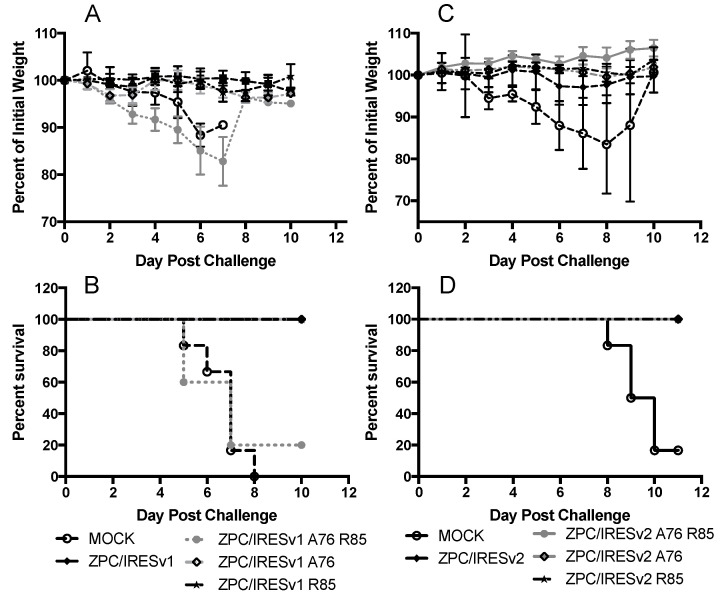
Mutant vaccine efficacy in adult CD1 mice. (**A**,**B**) show the percent of initial weight and survival from ZPC/IRESv1-derived vaccines, respectively. The same key is used for panels (**A**,**B**). (**C**,**D**) show the percent of initial weight and survival from ZPC/IRESv2-derived vaccines, respectively. Error bars denote standard error. The same key is used for panels (**C**,**D**).

The nonsynonymous E2 mutations (position 9300 as A76 and position 9327 as R85) were placed into the ZPC/IRESv1 and ZPC/IRESv2 infectious cDNA clones, either singly or in combination, then used to vaccinate 6–8-week-old CD1 mice to determine whether the vaccine’s phenotype has been altered (Figure 6 and Figure 7). All mice tolerated the vaccine well. Blood was taken on days 1 and 2 post vaccination to determine vaccine viremia (Figure 6). In general, the mutations incorporated into each ZPC/IRES vaccine did not alter the average viremia titers significantly except between parental ZPC/IRESv1 and ZPC/IRESv1 A76 (*p* > 0.05) or parental ZPC/IRESv2 and ZPC/IRESv2 A76 (*p* > 0.01) on day 1 or parental ZPC/IRESv1 and ZPC/IRESv1 A76 R85 (*p* > 0.5) on day 2. Interestingly, vaccines expressing the A76, and to a lesser extent the R85, mutation also generated a higher percentage of viremic mice compared to their parental vaccines.

Among all mutant vaccines, only the ZPC/IRESv1 A76/R85 mutant failed to protect against weight loss and a fatal outcome (Figure 7). Indeed, there was no statistical difference in survival between MOCK and ZPC/IRESv1 A76 R85 (Figure 7B, *p* = 0.75). ZPC/IRESv1 A76 R85-vaccinated mice also lost considerable weight with the exception of one mouse that maintained >90% of its weight during the same time period. All other vaccines fully protected mice from lethal challenge weight loss and morbidity.

Because neutralizing antibody levels directly correlate with protection from alphaviral disease, determining PRNT titers post vaccination is critically important. Sera from mice vaccinated once, either 4- or 20-weeks prior were analyzed for their ability to neutralize ZPC-738 (Table 2). Seroconversion was detected in the majority of ZPC/IRESv1 and ZPC/IRESv2 mice by week 4 with an average PRNT_80_ titer of 40 and 60.7, respectively. When tested on week 20, average titers rose but 100% seroconversion was detected only in the ZPC/IRESv2-vaccinated group. Vaccines with mutations yielded mixed immunogenicity by week 4; both A76 and R85 together or A76-alone in either ZPC/IRES vaccine reduced PRNT_80_ titers to undetectable or barely detectable levels. ZPC/IRES vaccines with the R85 mutation, however, retained their immunogenicity.

### 3.4. VEEV ZPC/IRES Are Immunogenic and Protective in Cynomolgus Macaques

Finally, to assess the immunogenicity and efficacy of ZPC/IRESv1 in the superior cynomolgus macaque model, five vaccinated animals (10^5^ PFU in a 0.1 mL volume) weighing 3–6 kg, and two MOCK (PBS)-vaccinated animals were bled prior to the study to confirm no prior immunity against VEEV. On day 35 post vaccination, all were bled and PRNT titers were detected in four of the five ZPC/IRESv1-vaccinated recipients (Table 3) albeit at lower levels than in mice. Expectedly, unvaccinated NHPs had no anti-VEEV neutralizing antibodies.

NHPs were challenged by aerosol exposure and monitored for changes in temperature and for viremia. The back-titrated aerosol doses ranged from 5.9–6.9 log_10_ pfu/NHP (mean 6.34 ± 0.35 pfu log_10_/NHP). Compared to pre-challenge baseline temperature data, both unvaccinated animals showed fever lasting for up to 6 days, with mean fever-hours of 145. In contrast, only 2 of 5 vaccinated animals developed detectable fever, with a mean of 75 fever-hours for this cohort. This difference in fever hours was significant (*p* < 0.01, one-tailed *t*-test). In addition to protection from disease, no vaccinated NHP had a detectable viremia for the first 3 days following challenge. Interestingly, NHP #3, which had no detectable PRNT titer, had a fever in the absence of a detectable viremia. NHP #5 had seroconverted and was protected against viremia but not fever.

A second set of NHPs was vaccinated with ZPC/IRESv2 to determine if the immunogenicity observed following ZPC/IRESv1 could be improved (Table 4). Here, NHPs were similarly vaccinated and challenged but not implanted with a telemetric temperature device. As a result, fever hours could not be calculated. Unlike ZPC/IRESv1 vaccination, all ZPC/IRESv2 vaccine recipients seroconverted. PRNT titers for all ZPC/IRESv2-vaccinated primates were also substantially higher than their ZPC/IRESv1-vaccinated counterparts, with a mean PRNT_80_ titer of 288. Further, all vaccinated NHPs were protected against VEEV strain 3908 viremia, although one sham-vaccinated NHP (#9) also failed to become viremic after aerosol challenge.

## 4. Discussion

There is a continued need and demand for safe and efficacious vaccines against the diseases caused by alphaviruses, including chikungunya, Mayaro, eastern and Venezuelan equine encephalitis viruses. We have shown for many of these viruses that the IRES platform addresses this need while also limiting their spread through mosquito vectors [22,24,35,36,37]. Here, we continue to improve upon and broaden the scope of our IRES-vaccines by including the ID serotype of VEEV.

In a previous study, we reported on the similar construction and evaluation of the IE subtype of VEEV IRES vaccines [24]. The 68U201/IRESv1 (VEEV/mutSG/IRES/1) and 68U201/IRESv2 (VEEV/IRES/C) both were able to elicit protective immunity in CD1 mice. Both vaccines also showed a marked decrease in neurovirulence in CD1 pups compared to wild type parent viruses, with little difference between them. The differentiation between these vaccines was apparent with the neutralizing antibody titers, as VEEV/IRES/C elicited two-fold greater PRNT_50_ and PRNT_80_ values in CD1 mice than VEEV/mutSG/IRES/1. A very similar trend is observed here with ZPC/IRESv1 and ZPC/IRESv2, where the latter produced higher PRNT_80_ titers, especially at 20-weeks-post vaccination (Table 3). More importantly, all ZPC/IRESv2-vaccinated mice produced a neutralizing antibody response after a single vaccination.

The decision to pursue ZPC/IRESv1 for characterization in the NHP model was made based upon the results from not only ZPC/IRES vaccine data, but also from other IRESv1 vaccines that have been heavily tested in mice and NHPs with positive results [25,38,39,40]. ZPC/IRESv1 was chosen as the first candidate because it provided protective murine immunity and had a similar safety profile to TC-83 in intracranially injected neonatal mice. The Version 2 vaccine based either the 68U201 [24] or TC-83 [22] VEEV backbones were also more virulent in previous studies. Five NHPs were vaccinated with ZPC/IRESv1 and 4 showed positive neutralizing antibody responses immediately prior to 3908 aerosol challenge. Interestingly, none of the vaccinated NHPs had a detectable viremia on days 1–3, even in the non-responder. However, the non-responder and two NHPs with a PRNT_80_ titer of 20 and 40, had 107, 110, or 10 fever-hours, respectively, suggesting that protection from disease was incomplete. The reason for this discordance between protection against viremia and fever is puzzling. The challenge virus used as the IC strain 3908 while the vaccine backbone was the ID strain ZPC-738. While the majority of vaccinated NHPs (and mice) did have heterologous cross-protection, there may be some incomplete protection at the lowest measurable PRNT_80_ titers. Further, viremia is not routinely measured as a correlate of protection, and human IND trials with the TC-83 vaccine have shown disconnects between adverse, flu-like symptoms, and immunogenicity [11]. Regardless, immunogenicity elicited by the ZPC/IRESv2 vaccine was superior to that of ZPC/IRESv1 in both mice and NHPs. CHIKV/IRESv2 also provided a better neutralizing antibody immune response than CHIKV/IRESv1 in cynomolgus macaques [40]. Furthermore, our earlier studies of the VEEV 68U201/IRESv2-vaccinated mice also had a slightly higher PRNT_80_ titers than those induced by 68U201/IRESv1, which lasted for more than a year after vaccination [24]. Unfortunately, due to the study design, fever-hour reductions could not be compared between ZPC/IRESv1 and ZPC/IRESv2.

One confounding issue in our challenge of the ZPC/IRESv2 NHPs was the inconsistent viremia in the sham-vaccinated group. This lack of consistent viremia makes it difficult to determine if the ZPC/IRESv2-vaccinated NHPs were truly protected from viremia or not adequately exposed to an appropriate dose of VEEV. Furthermore, fever-hours were not reported as part of this study so protection from disease could not be assessed either. It is likely that these NHPs would be protected from both viremia and fever based on their high PRNT_80_ titers (Table 4) but that conclusion cannot be reached from the data gathered in this experiment. However, experience from a similar study evaluating the efficacy of 68U201/IRESv1 showed complete protection from IE subtype 68U201-challenge in NHPs with a similar PRNT_80_ titer [25].

Serial passaging of ZPC/IRESv1 in Vero cells resulted in several consensus mutations, 3 of which were nonsynonymous changes to the E2 and E1 glycoproteins. When these E2 mutations, either in combination or alone, were genetically engineered back into the ZPC/IRESv1 vaccine, some constructs lost their ability to elicit a protective immune response. Surprisingly, ZPC/IRESv1 A76, R85 generated no vaccine-related viremia, and vaccinated mice were protected or had neutralizing antibody titers. When these mutations were investigated alone, vaccine-related viremia and PRNT_80_ titer were detected in only a portion of the mice but all were protected from lethal 3908 challenge. One explanation for this phenotype is the accumulation of positively charged amino acids on the E2 and E1 proteins that contribute to commonly seen cell culture adaptive mutations that facilitate binding to heparin sulfate (HS). Studies in Sindbis virus have long shown a negative correlation between HS binding and virulence [41,42], but this phenotype is more complex for VEEV [43]. Sindbis virus passaged on BHK-21 cells showed nonsynonymous mutations favoring positively charged amino acids like arginine that increased the ability of virions to bind to cells and a reduction in murine neonatal fatalities [42]. Likewise, Griffin and colleagues showed that the loss of positively charged amino acid changes in the E2 protein resulted in increased Sindbis virulence in sucking mice compared to those with more positive E2 proteins [41]. This is a common feature for serially passaged alphaviruses and underlines the importance of using low-passage stocks for studies. Additional experiments are required to determine when in the 10-passage series these mutations arose and whether genetically stabilizing this region using synonymous mutations can reduce the frequency of these mal-adaptive mutations. As it is highly unlikely that these mutations would arise naturally in vivo after vaccination, sera at peak viremia from these mutations were not sequenced. If a mutation did occur, it would likely be associated with a high viremia titer, which was not observed in these studies. Therefore, care would need to be taken to limit tissue culture amplification when creating vaccination stocks.

## 5. Conclusions

In summary, we have described the successful use of a vaccine based on the ZPC-738 virus containing an insertion of the IRES sequence to prevent VEE disease and/or VEEV infection. Both versions of ZPC/IRES elicited neutralizing antibodies in vaccinees, which is the main correlate of protection for preventing VEE disease, in both mice and nonhuman primates. We also identified mutations which may arise during serial passage of the vaccine which can influence vaccine efficacy.

## Figures and Tables

**Table 1:** Summary of consensus mutations following ZPC/IRESv1 passaging.

Position/Gene (Element)	4449/nsP2	7242/nsP4	7995/IRES	9300/E2	9327/E2	10925/E1
Wild-type *^a^*	GTA	GTG	A	GAG	CAC	GAA
Replicate #1	GTt *^b^*	GTa	T/A mix *^c^*	GcG	CgC	aAA
Replicate #2	GTt	GTa	T/A mix	GcG	CgC	aAA
Replicate #3	GTt	GTa	T/A mix	GcG	CgC	aAA
Mutation	Gly → Gly	Val → Val	n/a	Glu → Ala	His → Arg	Glu → Lys

*^a^* Unpassaged ZPC/IRESv1; *^b^* lower case letter indicates mutation; *^c^* chromatogram indicated mixed population at this position.

**Table 2:** PRNT_80_ neutralization titers following ZPC/IRES vaccination of mice.

Vaccine	PRNT_80_: 4-Weeks ^1^	% Group Seroconvert	N	PRNT_80_: 20-Weeks	% Group Seroconvert	N
ZPC/IRESv1	40 ± 42.4	11/15 (73.3%)	15	142.2 ± 225	5/8 (62.5%)	8
ZPC/IRESv2	60.7 ± 55.7	12/15 (80%)	15	324 ± 241.8	10/10 (100%)	10
ZPC/IRESv1 A76, R85	<LOD ^2^	1/10 (10%)	10	ND ^3^	-	-
ZPC/IRESv2 A76, R85	<LOD	0/10 (0%)	10	ND	-	-
ZPC/IRESv1 A76	31 ± 27	5/10 (50%)	10	ND	-	-
ZPC/IRESv2 A76	<LOD	1/10 (10%)	10	ND	-	-
ZPC/IRESV1 R85	172 ± 372	6/10 (60%)	10	ND	-	-
ZPC/IRESV2 R85	36 ± 46.5	4/10 (40%)	10	ND	-	-

^1^ average reciprocal titer ± Standard deviation; number of mice tested. ^2^ Average less than 20. ^3^ ND = not done.

**Table 3:** ZPC/IRESv1 vaccination and protection in cynomolgus macaques.

NHP #	Vaccination Status	PRNT_80_^1^ 35dpv	PRNT_50_ 35dpv	Viremia 1 dpi (log_10_ PFU/mL)^2^	Viremia 2 dpi (log_10_ PFU/mL) ^2^	Viremia 3 dpi (log_10_ PFU/mL) ^2^	Fever Hours
1	Sham-vaccinated	<20	<20	4.30	3.78	2	150
2	Sham-vaccinated	<20	<20	2.69	3.70	3.70	136
3	ZPC/IRESv1	<20	<20	<2	<2	<2	107
4	ZPC/IRESv1	<20	40	<2	<2	<2	10
5	ZPC/IRESv1	20	20	<2	<2	<2	110
6	ZPC/IRESv1	20	160	<2	<2	<2	0
7	ZPC/IRESv1	<20	20	<2	<2	<2	0

^1^ Reciprocal titer. LOD = 20. ^2^ Viremia titer LOD = 2 log_10_ pfu/mL serum. All samples less than the LOD are listed as <2.

**Table 4:** ZPC/IRESv2 vaccination and protection in cynomolgus macaques.

NHP #	Vaccination Status	PRNT_80_^1^ 28 dpv	PRNT_50_ 28 dpv	Viremia 1 dpi (log_10_ PFU/mL) ^2^	Viremia 2 dpi (log_10_ PFU/mL) ^2^	Viremia 3 dpi (log_10_ PFU/mL) ^2^
8	Sham-vaccinated	<20	<20	3.6	3.3	<1.3
9	Sham-vaccinated	<20	<20	<1.3	<1.3	<1.3
10	ZPC/IRESv2	320	>640	<1.3	<1.3	<1.3
11	ZPC/IRESv2	160	>640	<1.3	<1.3	<1.3
12	ZPC/IRESv2	320	>640	<1.3	<1.3	<1.3
13	ZPC/IRESv2	320	320	<1.3	<1.3	<1.3
14	ZPC/IRESv2	320	>640	<1.3	<1.3	<1.3

^1^ Reciprocal titer. ^2^ Viremia titer LOD = 1.3 Log_10_ PFU/mL. All samples less than the LOD are listed as <1.3.
